# The role of the large-conductance calcium-dependent potassium channel, BK/Slowpoke, in shaping motor neuron firing during rhythmic activity

**DOI:** 10.1186/1471-2202-12-S1-P217

**Published:** 2011-07-18

**Authors:** Maytee Cruz-Aponte, Adrian Smith, Marco A Herrera-Valdez, Erin C McKiernan

**Affiliations:** 1 Mathematical, Computational, and Modeling Sciences Center, Arizona State University, Tempe, AZ 85287, USA; 2 School of Life Sciences, Arizona State University, Tempe, AZ 85287, USA; 3 School of Human Evolution and Social Change, Arizona State University, Tempe, AZ 85287, USA

## 

Rhythmic muscle contractions underlie a number of crucial motor behaviors, such as respiration and locomotion. The timing of contractions is determined by the intrinsic activity and synaptic interactions of neurons within what are called central pattern generating (CPG) networks [1,2]. In many systems, motor neurons (MNs) are not part of the classically-defined CPG. However, research suggests that ionic currents in MNs may shape the timing of the final motor output [3,4]. A lot of work has focused particularly on the role of potassium currents in shaping responsiveness and firing of MNs [3,5]. Large-conductance calcium-dependent potassium (BK) currents, encoded by members of the *Slowpoke* (*Slo*) gene family, can contribute to action potential repolarization, regulation of firing frequency and interspike interval, repetitive firing, and burst termination [6]. Mutations of *Slo* genes also lead to a variety of motor disturbances [6]. We developed a biophysical model of bursting activity in MNs to explore the circumstances under which a BK/*Slo* current expressed in MNs can shape the timing of motor output underlying locomotion. We identify mechanisms by which the BK/*Slo* current changes the bursting output of MNs, and describe the different behaviors that are observed for varying membrane densities of the underlying channel. We also present preliminary data consisting of electrophysiological recordings from larval *Drosophila* showing that the changes in motor output predicted by the model are indeed observed when genetic manipulations of *Slo* channel density (RNA interference constructs) are targeted to MNs [7]. Our results not only further understanding of the specific role of BK/*Slo* channels in MNs, but contribute more generally to the growing knowledge on the role intrinsic MN properties play in shaping rhythmic motor output.
